# Supplementary material for: Environmental cues from neural crest derivatives act as metastatic triggers in an embryonic neuroblastoma model
Source: Nat Commun. 2022 May 10;13:2549. doi: 10.1038/s41467-022-30237-3 (PMC9091272; doi:10.1038/s41467-022-30237-3)
Supplement: Supplementary file 1 — Supplementary Information [file 41467_2022_30237_MOESM1_ESM.pdf]

Supplementary Information

Environmental cues from neural crest derivatives act as metastatic triggers in an embryonic neuroblastoma model

Dounia Ben Amar[1], Karine Thoinet[1], Benjamin Villalard[1], Olivier Imbaud[1], Clélia Costechareyre[2], Loraine Jarrosson[2], Florie Reynaud[1], Julia Novion Ducassou[3], Yohann Couté[3], Jean-François Brunet[4], Valérie Combaret[5], Nadège Corradini[6], Céline Delloye-Bourgeois[1*&], Valérie Castellani[1*&]

Supplementary Figures 1-9

Supplementary Tables 1

Supplementary References list

Supplementary Figure 1

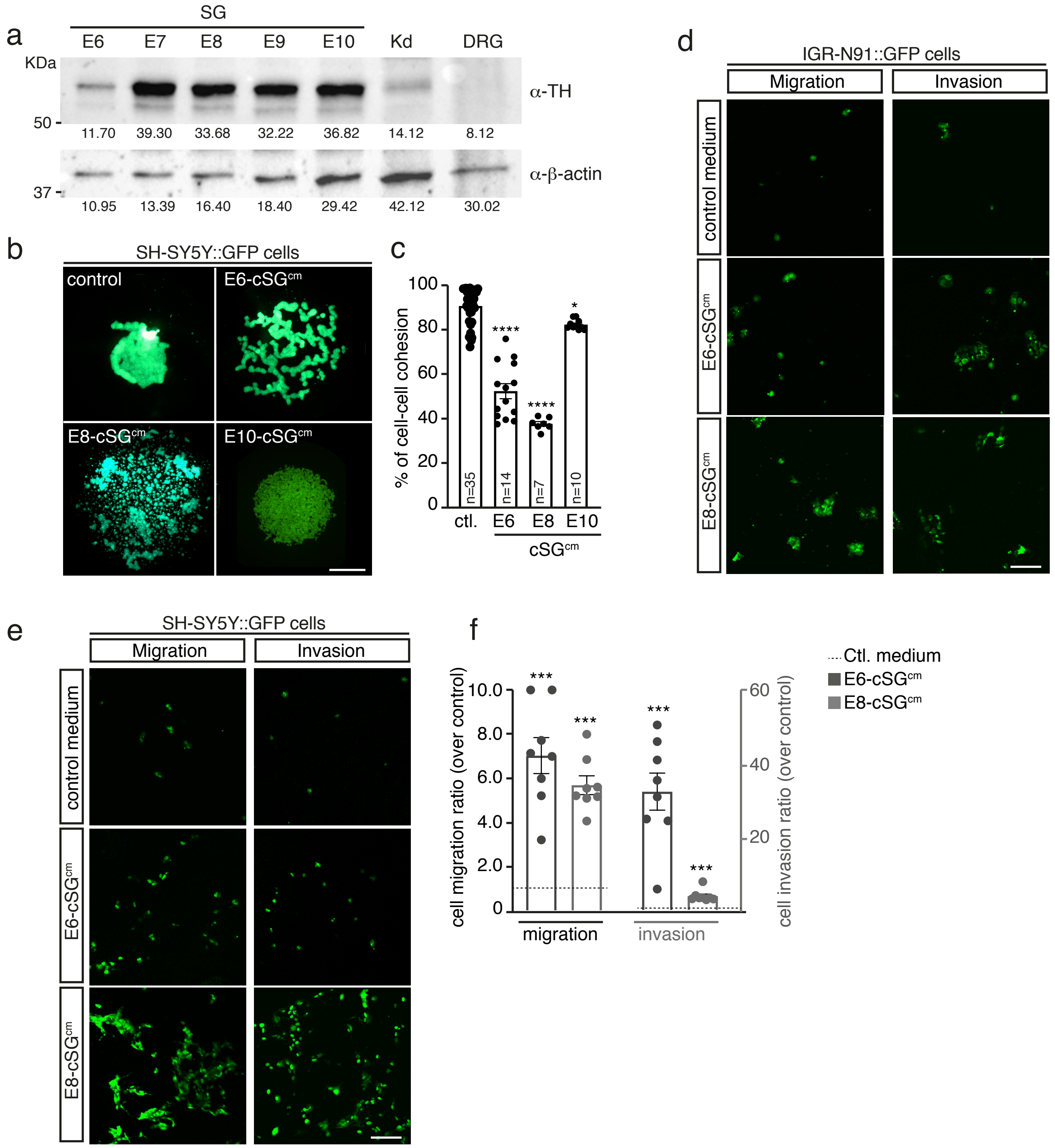

Supplementary figure 1: Sympathetic Ganglia conditioned medium (SG^{cm}) induces a decrease in neuroblastoma cell-cell cohesion and an increase of migration and invasion capacities. a. Representative western blots of Tyrosine Hydroxylase (TH) or ß-actin as loading control in E6 to E10 sympathetic ganglia, kidney (kd) and DRG (N=3 independent experiments). **b**. Representative pictures of SH-SY5Y cell aggregates treated with E6, E8 and E10-cSG^{cm}, compared to medium without any cultured tissue (control). Scale bar: 1 mm. **c**. Quantification of cell-cell aggregation rate for SH-SY5Y cells cultured in hanging drops and treated with E6, E8 and E10-cSG^{cm}, compared to medium without any cultured tissue (ctl.). (N=5 independent experiments; n: number of aggregates analyzed per condition; two-sided Mann-Whitney U test; comparison to control medium: E6-cSG^{cm}: $p<0.0001$, E8-cSG^{cm}: $p<0.0001$, E10-cSG^{cm}: $p=0.0110$). **d**. Representative pictures of IGR-N91 cells that migrated and invaded in transwell assays using E6 and E8-cSG^{cm} in the lower part of the device (N=7 independent experiments with E6-cSGcm, N=3 independent experiments with E8-cSGcm). Scale bar: 200 μm. **e**. Representative pictures of SH-SY5Y cells that migrated and invaded in transwell assays using E6 and E8-cS^{cm} in the lower part of the device. Scale bar: 200 μm. **f.** Quantification of SH-SY5Y cells migration and invasion properties in transwell assays using E6 and E8-cSG^{cm} in the lower part of the device. Ratios over the number of migrating/invading cells in the control medium condition (medium without any cultured tissue in the lower part) are shown. (N=4 independent experiments performed in duplicates; two-sided Mann-Whitney U test, comparison to control medium, migration/invasion: E6-cSGcm: p=0.0002/p=0.0002; E8-cSGcm: p=0.0002/p=0.0002). Error bars show SEM. Source data are provided as a Source Data file.

Supplementary Figure 2

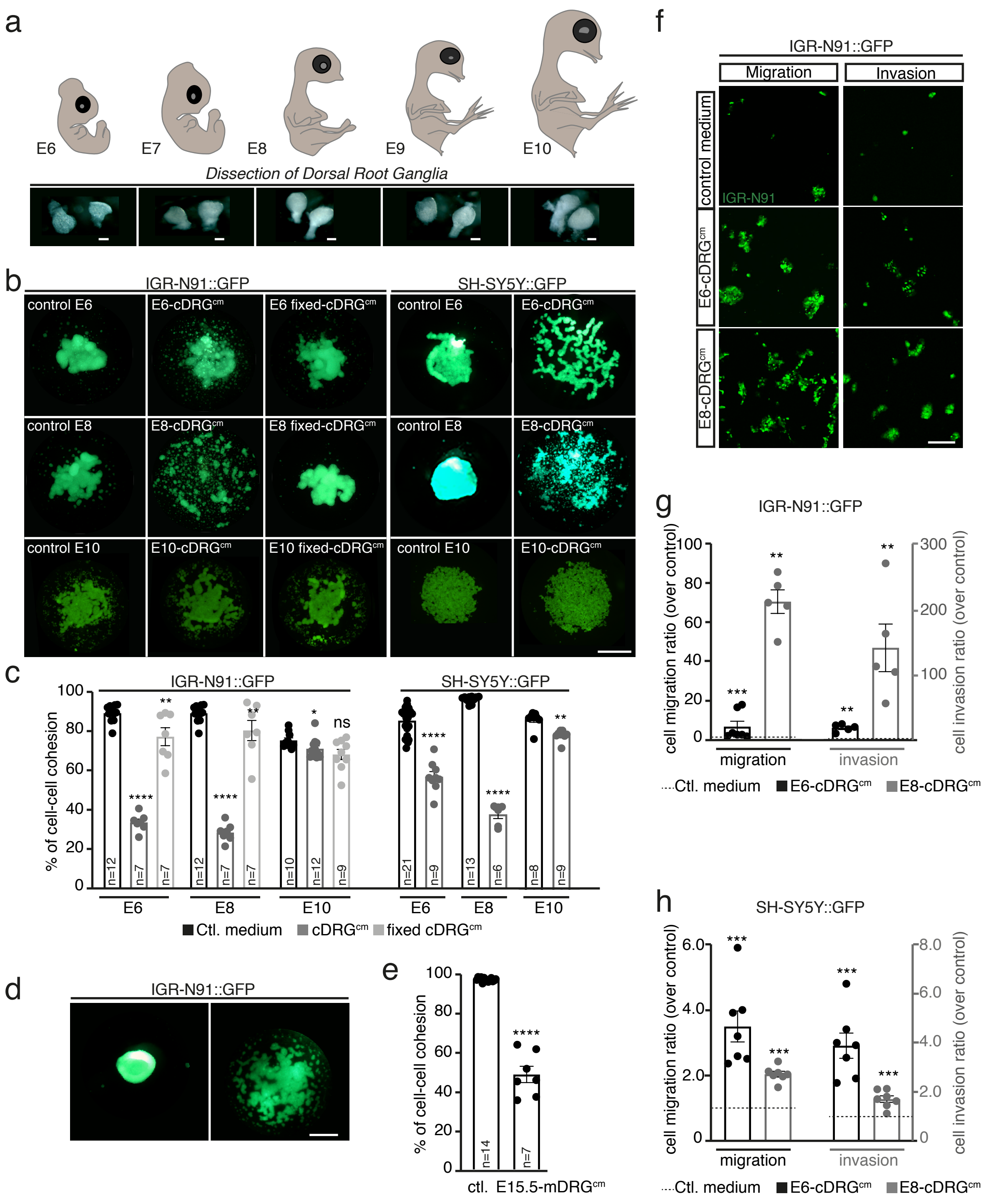

Supplementary figure 2: Dorsal root ganglia conditioned medium (DRGcm) induces a decrease in neuroblastoma cell-cell cohesion and an increase of migration and invasion abilities. a. Illustration of the dissection procedure of chick embryonic DRG from E6 to E10 developmental stages. Upper panels are schematic representations of chick embryos and lower panels show representative pictures of dissected DRG. Scale bar: 1 mm. **b**. Representative pictures of IGR-N91 and SH-SY5Y cell aggregates treated with E6-, E8- and E10-cDRG^{cm} prepared with living or fixed (fixed DRG^{cm}) DRG compared to medium without any cultured tissue (control). Scale bar: 1 mm. **c.** Quantification of cell-cell aggregation rate for IGR-N91 and SH-SY5Y cells cultured in hanging drops and treated with E6-, E8- and E10-cDRG^{cm}, prepared with live or fixed DRG compared to medium without any cultured tissue (ctl.) (N=4 independent experiments; n: number of aggregates analyzed per condition; two-sided Mann-Whitney U test; comparison to control medium in IGR-N91/SH-SY5Y cells: E6-, E8-, E10-cSGcm: p<0.0001/p<0.0001, p<0.0001/p<0.0001, p=0.0260/p=0.0049; E6-, E8-, E10-fixed-cS-Gcm: p=0.0072, p=0.0072, p=0.0563). **d.** Representative pictures of IGR-N-91 cell aggregates treated with E15.5-mDRG^{cm}, compared to medium without any cultured tissue (control). Scale bar: 1 mm. **e.** Quantification of cell-cell aggregation rate for IGR-N91 cells cultured in hanging drops and treated with E15.5-mDRG^{cm}, compared to medium without any cultured tissue (ctl.) (N=3 independent experiments; n: number of aggregates analyzed per condition; Mann-Whitney U test; p<0.0001). **f.** Representative pictures of IGR-N91 cells that migrated and invaded in transwell assays using E6 and E8-cDRG^{cm} in the lower part of the device. Scale bar: 200 μm. **g**. Quantification of IGR-N91 cells migration and invasion properties in transwell assays using E6 and E8-cDRG^{cm} in the lower part of the device. Ratios over the number of migrating/invading cells in the control condition (medium without any cultured tissue in the lower part) are shown (N=5 independent experiments, Mann-Whitney U test; comparison to control medium, migration/invasion: E6-cSGcm:

p=0.0006/p=0.0079; E8-cSGcm: p=0.0013/p=0.0079). **h**. Quantification of SH-SY5Y cells migration and invasion properties in transwell assays using E6 and E8-cDRG^{cm} in the lower part of the device. Ratios over the number of migrating/invading cells in the control condition (medium without any cultured tissue in the lower part) are shown (N=5 independent experiments, Mann-Whitney U test; comparison to control medium, migration/invasion: E6-cSGcm: p=0.0006/p=0.0006; E8-cSGcm: p=0.0006/p=0.0006). Error bars show SEM. Source data are provided as a Source Data file.

Supplementary Figure 3

a

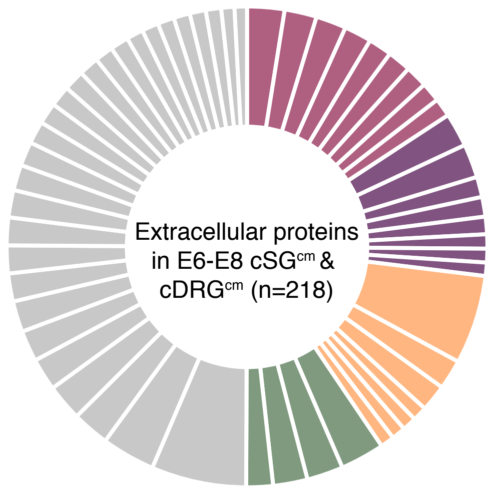

b

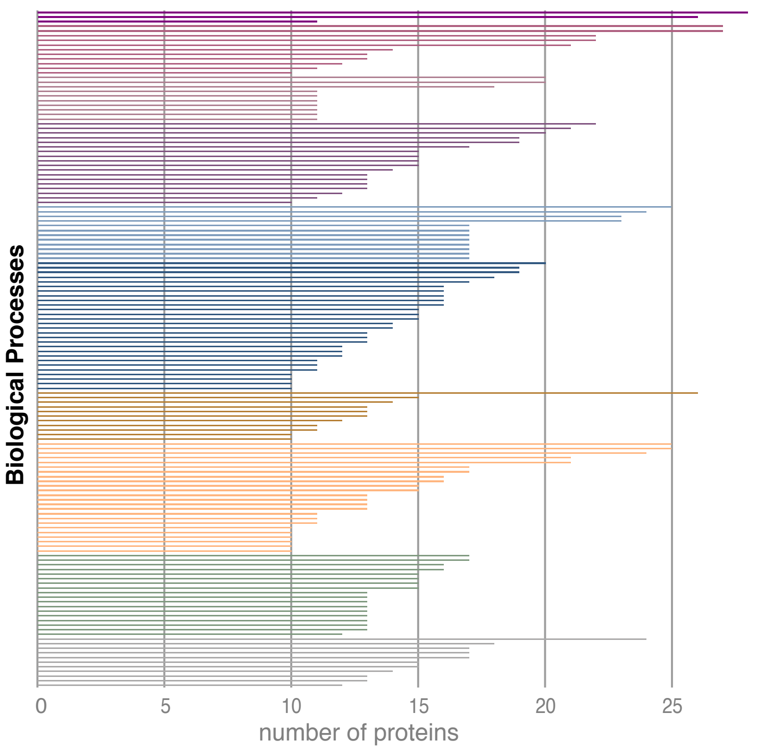

c

Protein	References
CDH11	Vallin 1998 (19); McCusker 2009 (20)
CDH2	Hatta 1986 (21); Akitaya 1992 (22); Nakagawa 1998 (23)
COL1A2	Lallier 1992 (24)
FN1	Wang 2016 (25)
MARCKS	Ruiz-Perera 2013 (26)
MMP2	Cai 2000 (27); Duong 2004 (28)

Protein	References
OLFM1	Barembaum 2000 (30); Moreno 2001 (29)
OLFML2A	Tomarev 2009 (16)
SPON1	Debby-Brafman 1999 (31)
TGFBI	Kawakami 2002 (32); Li 2020 (33)
TIMP2	Duong 2004 (28); Cantemir 2004 (34)
VCAN	Perris 1996 (35); Dutt 2006 (36)

Supplementary Figure 3: **a**. GO biological processes significantly represented in extracellular proteins present in cSGcm and cDRGcm at E6 and E8 stages. Each condition was duplicated to perform RNASeq (n=218 proteins; N>5 hits in each biological process related-gene set; hypergeometric test, p<0.01). Biological processes are color-coded according to 4 major classes: Cell movement & migration; Response to extracellular cues & signaling; Metabolism; Immune signaling. Sectors thickness represents the proportion of hits detected in each GO biological process related-gene set. **b**. GO biological processes significantly represented in extracellular proteins present in cSGcm and cDRGcm at E6 and E8 stages but not or barely detected in E10 chick conditioned media (n=108 proteins; N>10 hits in each biological process related-gene set; hypergeometric test, p<0.01). **c**. Listed litterature linking proteins detected in conditioned media to neural-crest related processes.

Supplementary Figure 4

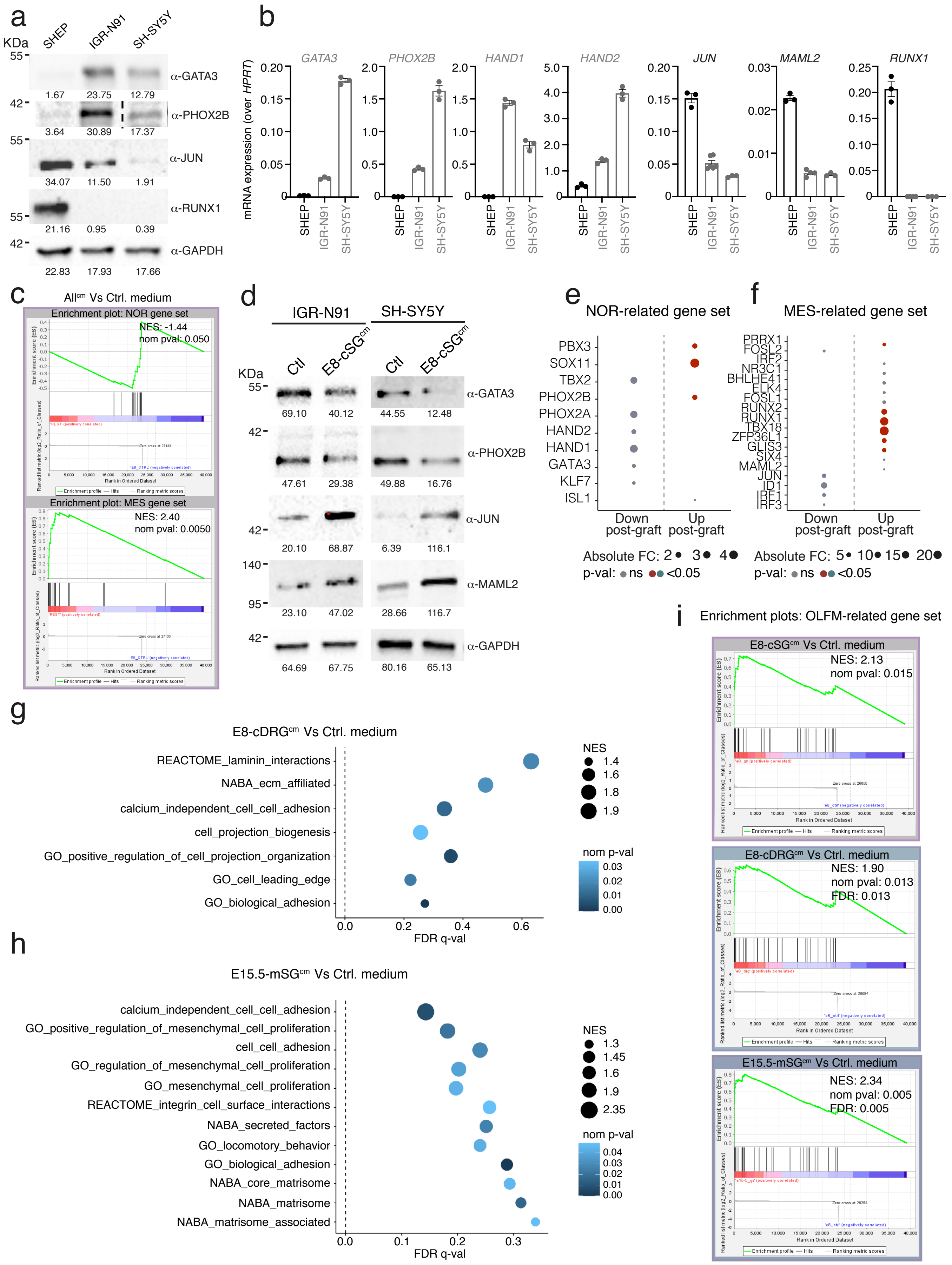

Supplementary figure 4: Paraspinal secreted cues trigger NB cells shift in gene programs involved in cell cohesion and motility. a. Representative western blot analysis of key NOR (GATA3, PHOX2B) and MES (JUN, RUNX1) proteins expression in SHEP, SH-SY5Y and IGR-N91 NB cell lines. The samples derive from the same experiment, blots were processed in parallel. Densitometric quantifications are indicated below each band of interest (N=3 independent experiments). **b.** qPCR quantification of mRNA expression of key NOR (GATA3, PHOX2B, HAND1, HAND2) and MES (JUN, MAML2, RUNX1) transcripts in SHEP, SH-SY5Y and IGR-N91 NB cell lines (N=3 independent experiments, Mann-Whitney U test). Error bars show SEM. **c.** Enrichment plots used to calculate the enrichment score of NOR-related (upper panel) and MES-related (lower panel) gene sets in all conditioned media-treated IGR-N91 cell aggregates compared to the control condition (phenotype-based permutation test, nom p-val <0.05; RNASeq samples were duplicated). **d.** Representative western blot analysis of key NOR (GATA3, PHOX2B) and MES (JUN, MAML2) proteins expression in IGR-N91 and SH-SY5Y NB cell lines incubated or not for 24 hours with E8-cSG-^{cm}. The samples derive from the same experiment, blots were processed in parallel. Densitometric quantifications are indicated below each band of interest (N=2 independent experiments). **e,f.** Comparative expression of NOR- (d) and MES- (e) related transcripts in non-grafted IGR-N91 cells and in IGR-N91-derived tumors formed in sympathetic ganglia 2 days after their graft in the avian trunk neural crest (GSE91377, published in Delloye-Bourgeois et al, 2017)**.** **g.** Gene Set Enrichment Analysis (GSEA) of a collection of 7 gene signatures related to cell motile behaviors in E8-cDRG^{cm}-treated IGR-N91 cell aggregates compared to control. Significantly regulated gene signatures (NES>1.3; phenotype-based permutation test, nom p-val<0.05) are outlined in the table. **h**. Gene Set Enrichment Analysis (GSEA) of a collection of 12 gene signatures related to cell motile behaviors in E15.5-mSG^{cm}-treated IGR-N91 cell aggregates compared to control. Significantly regulated gene signatures (NES>1.3; phenotype-based permutation test, nom p-val<0.05) are outlined in the table. **i**. Enrichment plots and scores (GSEA) analyzing the behavior of OLFM-related gene set in E8-cSG^{cm} (upper panel), E8-cDRG^{cm} (middle panel) and E15-mSG^{cm} (lower panel) versus control medium-treated IGR-N91 cell aggregates (phenotype-based permutation test, nom p-val <0.05). Source data are provided as a Source Data file.

Supplementary Figure 5

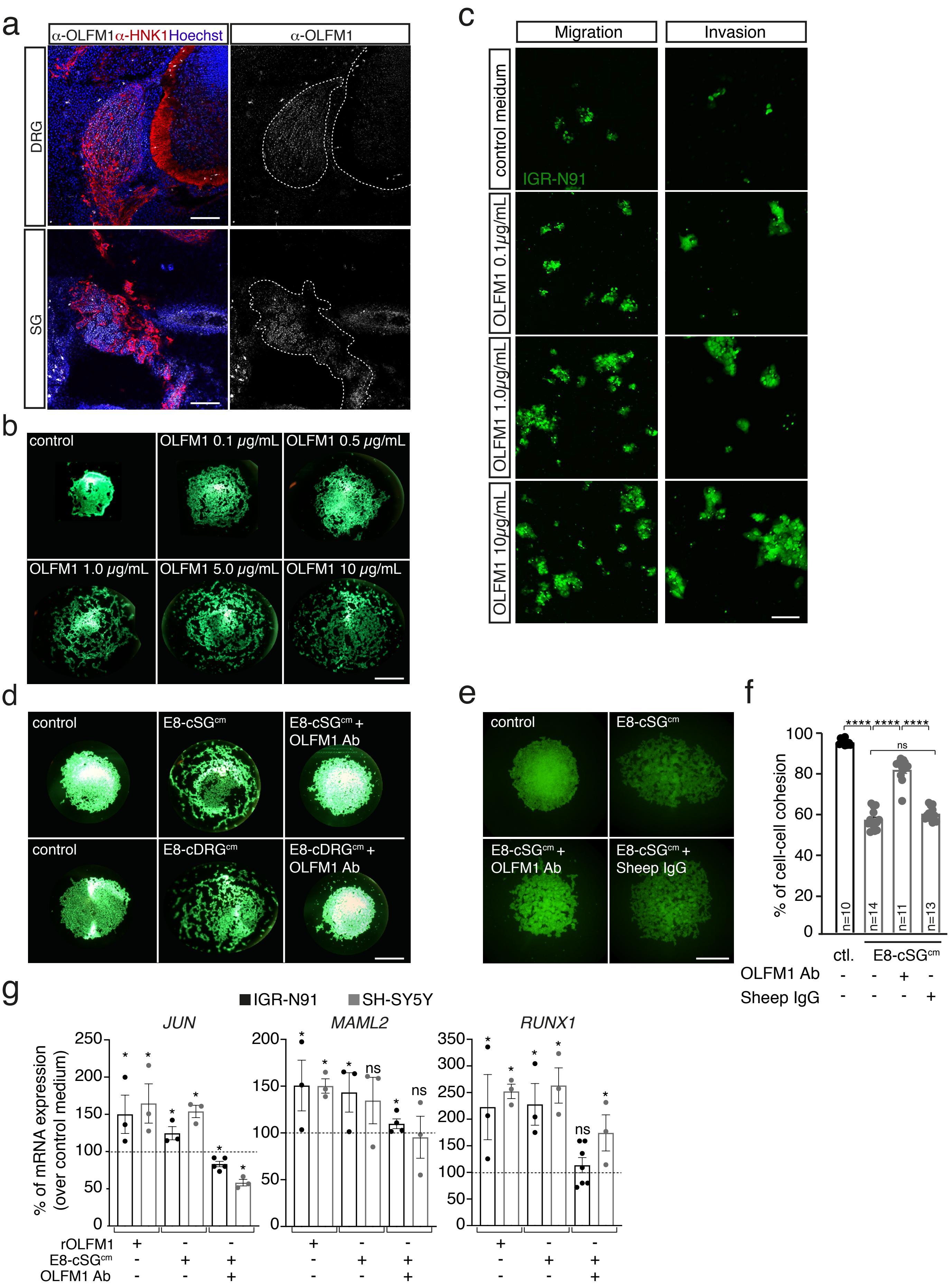

Supplementary figure 5: Paraspinal-derived OLFM1 induces a decrease in neuroblastoma cell-cell cohesion and an increase of migration and invasion capacities. a. Representative pictures of OLFM1 protein detection by immunofluorescence (in grey) in E6 chick embryo transverse sections, with a focus in the DRG (upper panels) and in the SG (lower panels). Neural crest derived cells were labeled with HNK1 immunofluorescence (in red); nuclei were labeled with Hoeschst (in blue) (n=5 E6 embryos). Scale bar: 100 μm. **b.** Representative pictures of IGR-N91 cell aggregates treated with increasing doses of recombinant OLFM1 (rOLFM1), compared to control medium (ctl.) (N=5 independent experiments). Scale bar: 1 mm. **c.** Representative pictures of IGR-N91 cells that migrated and invaded in transwell assays using increasing doses of recombinant OLFM1 (rOLFM1), in the lower part of the device (N=7 and N=10 independent experiments for migration and invasion assays). Scale bar: 200 µm. **d.** Representative pictures of IGR-N91 cell aggregates treated with E8-cSG^{cm} or E8-cDRG^{cm} supplemented or not with OLFM1 blocking antibody (OLFM1 Ab) (N=4 independent experiments). Scale bar: 1 mm. **e,f.** Representative pictures (e) and quantification of cell-cell cohestion rate (f) of IGR-N91 cell aggregates treated with E8-cSG^{cm} supplemented or not with 10 μg/mL of OLFM1 blocking antibody (OLFM1 Ab) or an isotypic control antibody (Sheep IgG) (N=4 independent experiments; n: number of aggregates analyzed per condition; two-sided Mann-Whitney U test; ctl vs E8-cSG^{cm}: p<0.0001, E8-cSG^{cm} vs E8-cSG^{cm} + OLFM1 Ab: p<0.0001, E8-cSG^{cm} + OLFM1 Ab vs E8-cSG^{cm} + Sheep IgG: p<0.0001, E8-cSG^{cm} vs E8-cSG^{cm} + Sheep IgG: p=0.0660). Error bars show SEM. **g.** mRNA quantification of key MES-related genes (JUN, MAML2 and RUNX1) in IGR-N91 and SH-SY5Y cell lines incubated either with 10 µg/mL rOLFM1 or E8-cSG^{cm} supplemented or not with OLFM1 Ab. Results are presented as a ratio over mRNA quantifications in the control condition (N=3 to 6 independent experiments; one-sided Mann-Whitney test; comparison to control IGR-N91/SH-SY5Y: JUN, rOLFM1: p=0.049/p=0.049, E8-cSG^{cm}: p=0.049/p=0.049, E8-cSG^{cm} + OLFM1 Ab: p=0.0179/p=0.049; MAML2, rOLFM1: p=0.049/p=0.049, E8-cSG^{cm}: p=0.049/p=0.350, E8-cSG^{cm} + OLFM1 Ab: p=0.0286/p=0.3500; RUNX1: rOLFM1: p=0.049/p=0.049, E8-cSG^{cm}: p=0.049/p=0.049, E8-cSG^{cm} + OLFM1 Ab: p=0.6667/p=0.049.Error bars show SEM. Source data are provided as a Source Data file.

Supplementary Figure 6

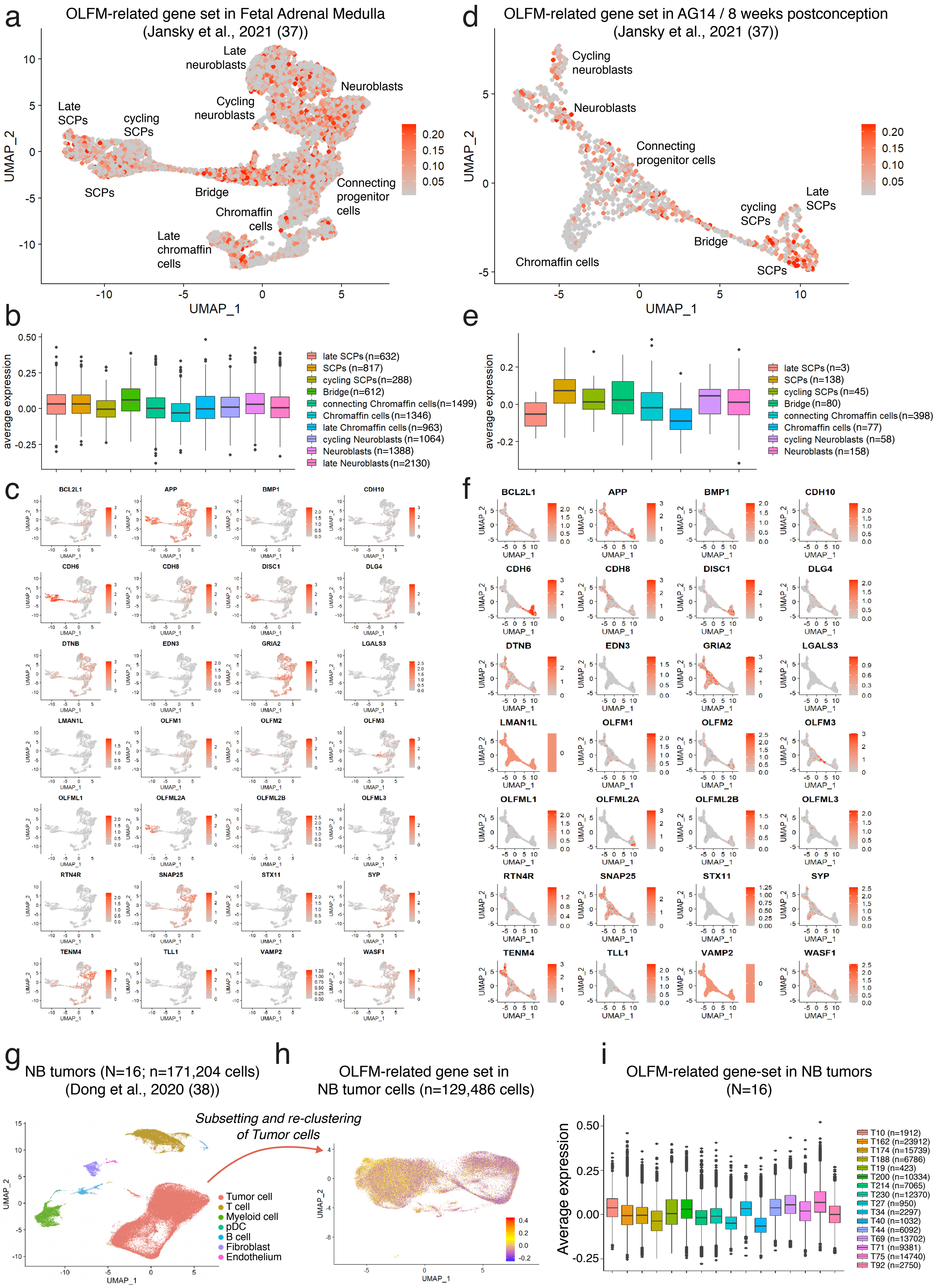

Supplementary figure 6: OLFM-related gene set is heterogeneously expressed in fetal adrenal medullary gland lineages and in NB tumor cells. a-c. Analysis of the expression of OLFM-related gene set in single cell RNASeq data of fetal adrenal medulla published by Jansky et al. (2021). In UMAP embedding of adrenal medullary cells (**a**), clusters were reannotated following the same approach than Jansky et al. The color indicates log-normalized gene expression. The average expression of OLFM-related gene set in each annotated cluster is shown in (**b**). In **c**, expression of each gene of the OLFM-related gene set is shown in UMAP embedding of fetal adrenal medullary cells. **d-f.** Analysis of the expression of OLFM-related gene set in single cell RNASeq data of early fetal adrenal medulla (AG14-8 weeks post conception) published by Jansky et al. (2021). In UMAP embedding of AG14 cells (**d**), clusters were reannotated following the same approach than Jansky et al. The color indicates log-normalized gene expression. The average expression of OLFM-related gene set in each annotated cluster is shown in (**e**). In **f**, expression of each gene of the OLFM-related gene set is shown in UMAP embedding of AG14 cells. **g-i**. Analysis of the expression of OLFM-related gene set in single cell RNASeq data of 16 NB tumors published by Dong et al. (2020). In UMAP embedding of all tumors (**g**), tumor cells cluster was identified as in Dong et al., according to the expression of a neuroendocrine (NE) gene set. Cells identified as tumor cells were subsetted and re-clustered in (**h**). The average expression of OLFM-related gene set in this UMAP embedding is shown in (**h**). The color indicates log-normalized gene expression. In **i**, the average expression of OLFM-related gene set in each NB tumor is shown. For box plots in b, e, i, the midline is the median, with the upper and lower limits of the box being the third and first quartile1 (75th and 25th percentile) respectively. The whiskers extend up to 1.5 times the interquartile range from the top (bottom) of the box to the furthest datum within that distance. Data beyond that distance are represented individually as points.

Supplementary Figure 7

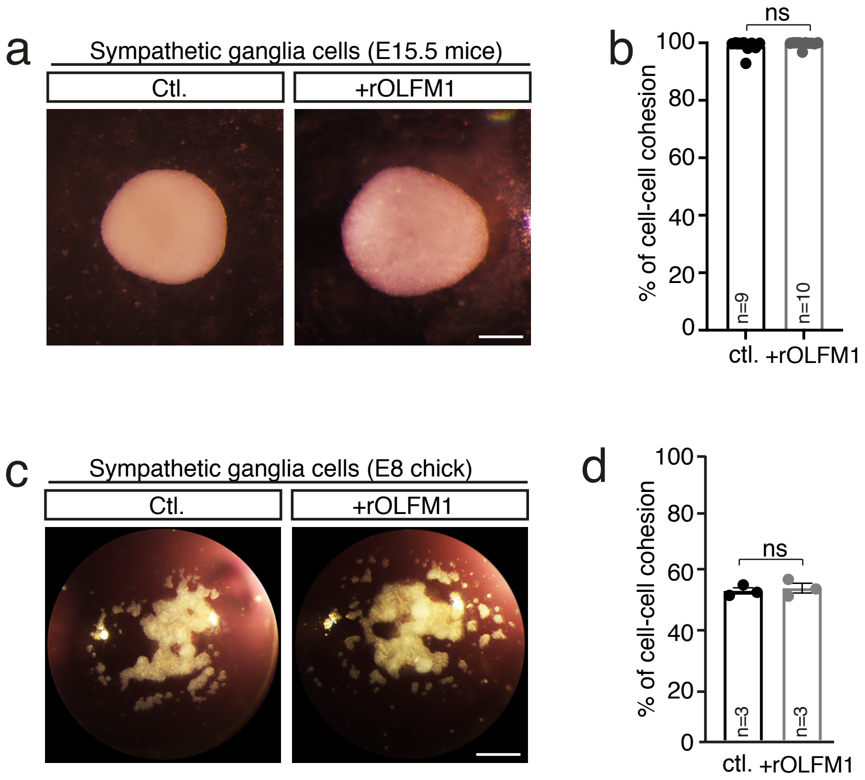

Supplementary figure 7: Recombinant OLFM1 doesn't affect sympathetic ganglia cell-cell cohesion. a. Representative pictures of cell aggregates obtained from dissociated E15.5 mouse sympathetic ganglia treated with recombinant OLFM1 (rOLFM1). Scale bar: 1 mm. **b**. Quantification of cell-cell cohesion rate for dissociated cells from E15.5 mouse sympathetic ganglionic chains, cultured in hanging drops and treated with recombinant rOLFM1 compared to control medium (ctl.) (N=3 independent experiments; n: number of aggregates analyzed per condition; two-sided unpaired t test; p=0.3221).**c.** Representative pictures of cell aggregates obtained from dissociated E8 chick sympathetic ganglia treated with recombinant OLFM1 (rOLFM1). Scale bar: 1 mm. **d**. Quantification of cell-cell cohesion rate for dissociated cells from E8 chick sympathetic ganglionic chains, cultured in hanging drops and treated with recombinant rOLFM1 compared to control medium (ctl.) (N=3 independent experiments; n: number of aggregates analyzed per condition; two-sided Mann-Whitney test; p=0.9999). Error bars show SEM. ns: not significant. Source data are provided as a Source Data file.

Supplementary Figure 8

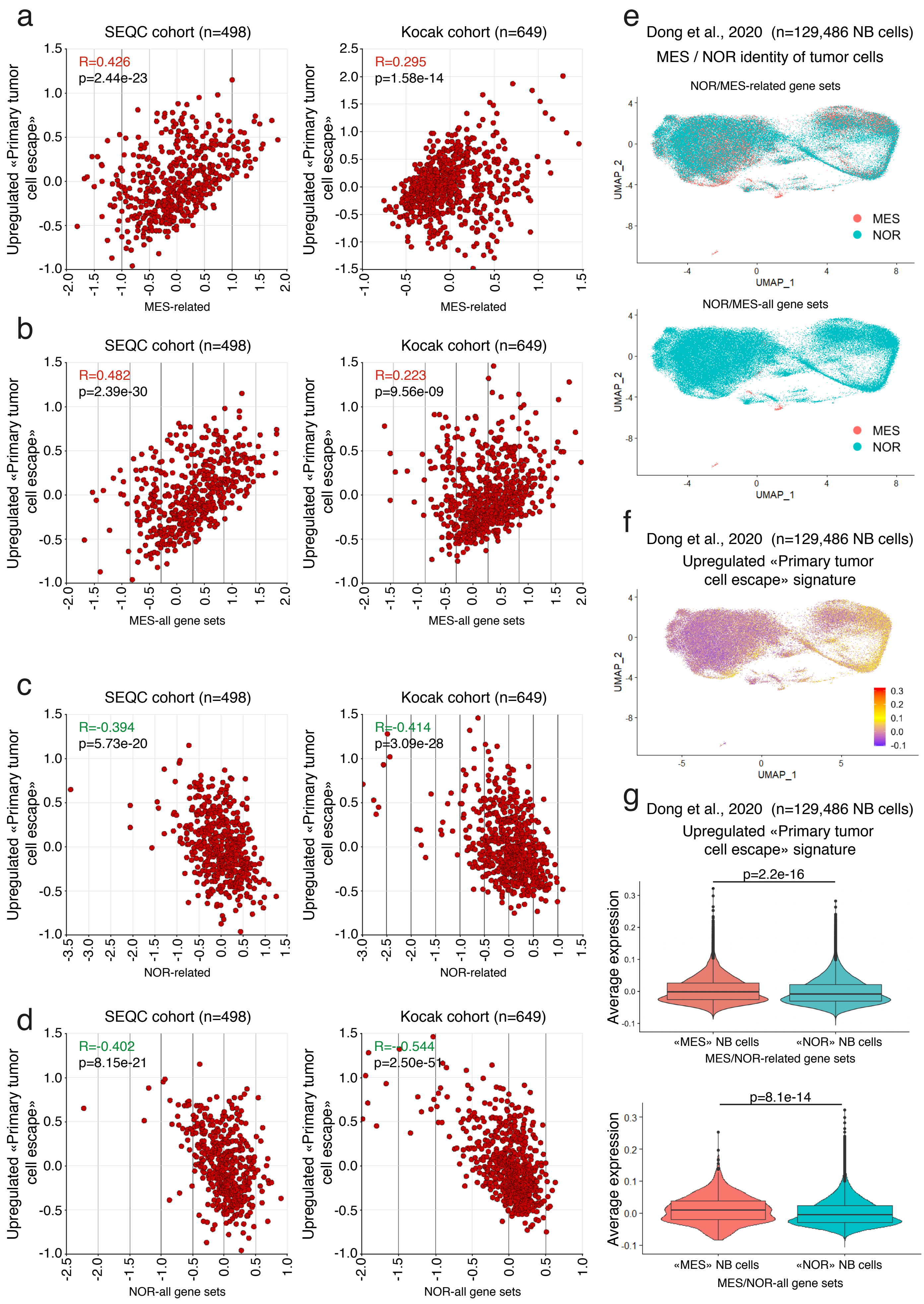

Supplementary figure 8: Genes upregulated upon NB primary tumor escape positively correlate with MES-related gene sets and negatively correlate with NOR-related gene sets. a,b. Correlation between genes upregulated in NB «Primary tumor escape» and gene sets related to a MES phenotype (a restricted MES-related gene set (MES-related, a) or a global MES gene set associating published MES/NCC-related gene sets (MES-all, b) in both Shi and Fisher's (GSE62564; n=498 samples, left panels) and Kocak's (GSE45547; n=649 samples, right panels) cohorts. **c,d.** Correlation between genes upregulated in NB «Primary tumor cell escape» and gene sets related to a NOR phenotype (a restricted NOR-related gene set (NOR-related, a) or a global NOR gene set associating published NOR/ADR-related gene sets (NOR-all, b) in both Shi and Fisher's (GSE62564; n=498 samples, left panels) and Kocak's (GSE45547; n=649 samples, right panels) cohorts. Average z-score values over genes within the respective gene sets were computed and used to relate gene signatures based on Fisher's exact test. Correlation coefficients and exact p-values are indicated on each graph. **e.** Feature plot assigning MES or NOR identity to NB tumor cells in Dong et al. scRNASeq dataset - see Supp. Fig. 6gh for initial clustering - , according to the highest NOR or MES score using either NOR/MES-related gene sets (upper panel) or NOR/MES-all gene sets (lower panel). **f.** Feature plot of the average expression score of genes upregulated in «Primary tumor cell escape» signature in NB tumor cells from Dong et al. scRNASeq dataset. **g.** Violin plot comparing the expression of genes upregulated in «Primary tumor cell escape» in NB cells having MES or NOR assigned identities, according to the expression of MES/NOR-related gene sets (upper panel) or MES/NOR-all gene sets. Two-sided unpaired t-test.

Supplementary Figure 9

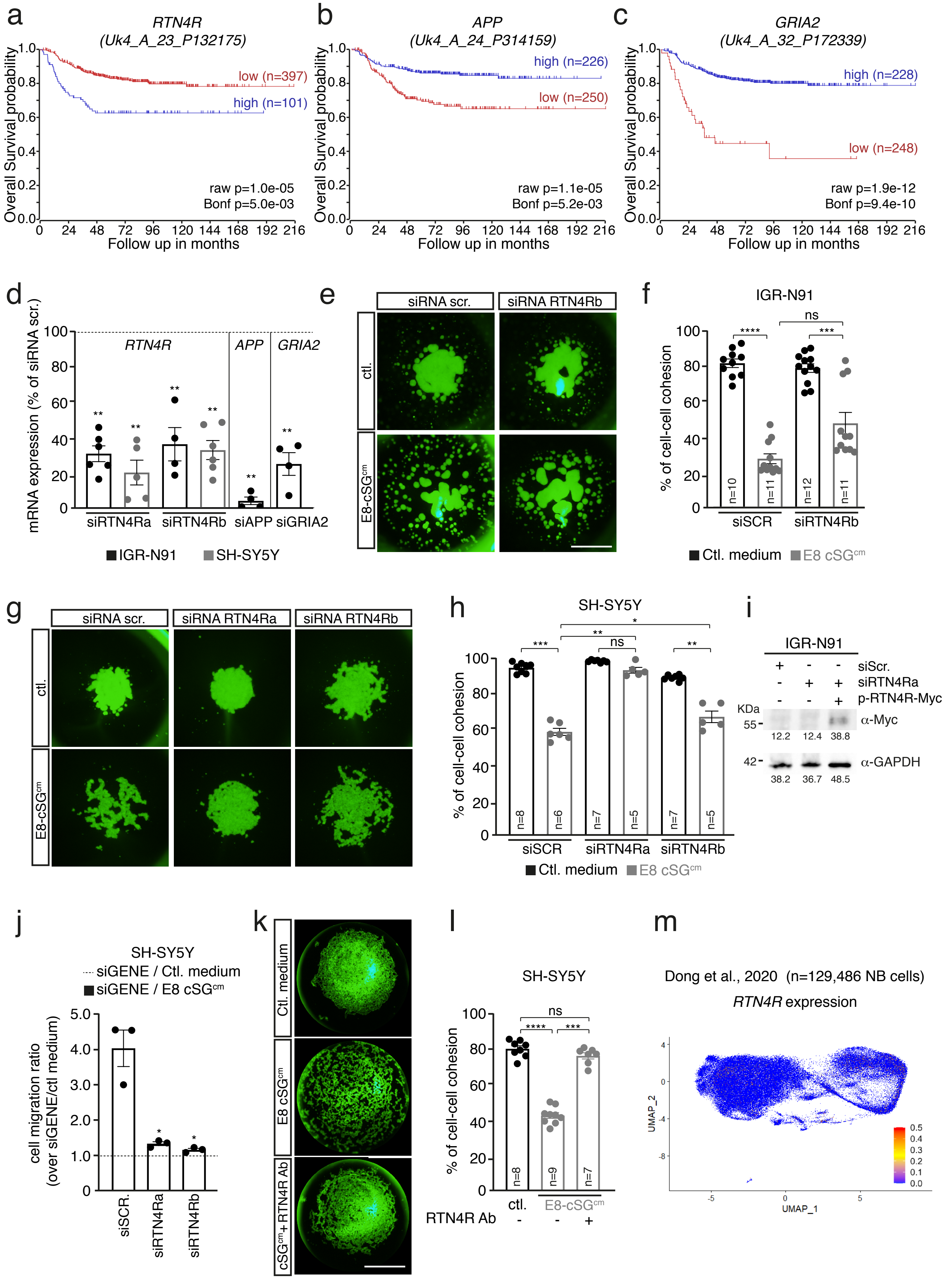

Supplementary figure 9: RTN4R is functionally involved in NB loss of cell-cell cohesion. a-c. Kaplan-Meier analysis of overall survival probability according to RTN4R (a), APP (b) and GRIA2 (c) expression levels in Kocak and collaborators published cohort (GEO: GSE45547; Kocak et al., 2013; http://r2.amc.nl; n = 649 samples; log rank test, raw and Bonferroni corrected p values are indicated on the graphs. **d**. mRNA quantification of RTN4R, APP and GRIA2, 48h after transfection of a control siRNA (siRNA scr.), or 2 different RTN4R siRNAs (siRTN4Ra, siRTN4Rb), or APP siRNA (siAPP) or GRIA2 siRNA (siGRIA2) in IGR-N91 and SH-SY5Y cell lines. Data are presented as a mean ratio over the control siRNA condition (siRNA scr.). HPRT was used as a housekeeping gene (N=4 to 6 independent experiments; two-sided Mann-Whitney U test; comparison to siScr IGR-N91/SH-SY5Y: siRTN4Ra:p=0.0022/p=0.0022; siRTN4Rb: p=0.0048/p=0.0022; siAPP: p=0.0048; siGRIA2: p=0.0048). Error bars show SEM. **e,f**. Representative pictures (e) and quantification of cell-cell cohesion rate (f) of IGR-N91 cell aggregates transfected with a control (siSCR) or a RTN4R siRNA (siRTN4Rb) and treated with E8-cSG^{cm} (N=4 independent experiments; n: number of aggregates analyzed per condition; two-sided Mann-Whitney U test; ctl vs E8-cSGcm: siScr: p<0.0001, siRTN4Rb: p=0.0008; E8-cSGcm/siScr vs E8-cSGcm/siRTN4Rb: p=0.0041. Error bars show SEM. **g,h**. Representative pictures (g) and quantification of cell-cell cohesion rate (h) of SH-SY5Y cell aggregates transfected with a control (siSCR) or two different RTN4R siRNAs (siRTN4Ra, siRTN4Rb) and treated with E8-cSG^{cm} (N=4 independent experiments; n: number of aggregates analyzed per condition; two-sided Mann-Whitney U test; ctl vs E8-cSGcm: siScr: p=0.0007, siRTN4Ra: p=0.0808, siRTN4Rb: p=0.0025; comparison to E8-cSGcm/siScr: E8-cSGcm/siRTN4Ra: p=0.0043, E8-cSGcm/siRTN4Rb: p=0.0779. Error bars show SEM. **i.** Western blot analysis of IGR-N91 cells transfected with human RTN4R expression plasmid; RTN4R is tagged with a myc epitope.The samples derive from the same experiment, blots were processed in parallel. Densitometric quantifications are indicated below each band of interest (N=3 independent experiments). **j.** Quantification of SH-SY5Y cells migration properties in transwell assays using E8-cSG^{cm} in the lower part of the device. Cells were transfected either with a control (siSCR) or two different RTN4R siRNAs (siRTN4Ra, siRTN4Rb). Ratios over the number of migrating cells in the control condition (medium without any cultured tissue in the lower part) are shown (N=3 independent experiments; one-sided Mann-Whitney U test; comparison to siScr: siRTN4Ra: p=0.049, siRTN4Rb: p=0.049). Error bars show SEM. **k,l**. Representative pictures (k) and quantification of cell-cell cohesion rate (l) of SH-SY5Y cell aggregates treated with E8-cSG^{cm} supplemented or not with 50 µg/mL RTN4R antibody (RTN4R Ab) (N=3 independent experiments; n: number of aggregates analyzed per condition; two-sided Mann-Whitney U test; ctl vs E8-cSGcm: p<0.0001, E8-cSGcm vs E8-cSGcm + RTN4R Ab: p=0.0002, ctl vs E8-cSGcm + RTN4R Ab: p=0.1520. Error bars show SEM. Scale bar: 1 mm. **m.** Feature plot of RTN4R gene expression in single cell RNASeq dataset of 129,486 NB tumor cells, curated from Dong et al. (2020). Source data are provided as a Source Data file.

Supplementary Table 1

term_goid	term_name	p-value	q-value	Effective_ size	Nb of items in query	Ratio
GO:1990748	cellular detoxification	3,91E-09	2,27E-08	109	12	11,0%
GO:0032963	collagen metabolic process	1,50E-07	5,82E-07	97	10	10,3%
GO:0006457	protein folding	3,12E-07	1,17E-06	226	14	6,2%
GO:0019882	antigen processing and presentation	1,84E-04	5,22E-04	176	9	5,1%
GO:0007163	establishment or maintenance of cell polarity	6,26E-04	1,60E-03	208	9	4,3%
GO:0001503	ossification	5,60E-06	1,94E-05	372	16	4,3%
GO:0032940	secretion by cell	1,01E-20	6,45E-19	1489	63	4,2%
GO:0002252	immune effector process	1,57E-17	6,70E-16	1284	54	4,2%
GO:0045321	leukocyte activation	1,43E-15	2,62E-14	1281	51	4,0%
GO:0001775	cell activation	8,81E-17	2,25E-15	1432	56	3,9%
GO:0008037	cell recognition	1,31E-03	3,22E-03	231	9	3,9%
GO:0055114	oxidation-reduction process	1,33E-10	1,00E-09	991	37	3,7%
GO:0030029	actin filament-based process	5,89E-08	2,60E-07	726	27	3,7%
GO:0044706	multi-multicellular organism process	3,88E-03	8,70E-03	222	8	3,6%
GO:0007568	aging	7,83E-04	1,96E-03	307	11	3,6%
GO:0090130	tissue migration	1,53E-03	3,70E-03	284	10	3,5%
GO:0009056	catabolic process	9,47E-24	1,21E-21	2542	88	3,5%
GO:0044419	interspecies interaction between organisms	8,94E-07	3,18E-06	833	27	3,2%
GO:0009628	response to abiotic stimulus	7,43E-09	3,97E-08	1148	37	3,2%
GO:0050817	coagulation	1,86E-03	4,40E-03	342	11	3,2%
GO:0007155	cell adhesion	3,48E-10	2,48E-09	1387	44	3,2%
GO:0048589	developmental growth	3,48E-05	1,06E-04	631	20	3,2%
GO:0006955	immune response	7,52E-16	1,60E-14	2252	70	3,1%
GO:0044281	small molecule metabolic process	8,14E-14	8,01E-13	2006	62	3,1%
GO:0009719	response to endogenous stimulus	4,82E-10	3,09E-09	1622	48	3,0%
GO:0016049	cell growth	2,66E-03	6,07E-03	467	13	2,8%
GO:0048870	cell motility	1,06E-07	4,25E-07	1618	44	2,7%
GO:0051674	localization of cell	1,06E-07	4,25E-07	1618	44	2,7%
GO:0048646	anatomical structure formation involved in morphoger	3,39E-05	1,06E-04	1075	28	2,6%
GO:0006928	movement of cell or subcellular component	2,41E-08	1,14E-07	2074	53	2,6%
GO:0061919	process utilizing autophagic mechanism	9,33E-03	1,99E-02	484	12	2,5%
GO:0065008	regulation of biological quality	1,42E-14	2,02E-13	3910	94	2,4%
GO:0009653	anatomical structure morphogenesis	6,13E-09	3,41E-08	2554	61	2,4%
GO:0042221	response to chemical	8,49E-17	2,25E-15	4644	109	2,3%
GO:0033036	macromolecule localization	1,27E-09	7,76E-09	3036	70	2,3%
GO:0006950	response to stress	2,18E-12	1,75E-11	3958	90	2,3%
GO:0051641	cellular localization	1,28E-08	6,55E-08	2867	65	2,3%
GO:0051234	establishment of localization	2,08E-14	2,67E-13	5224	112	2,1%
GO:0019725	cellular homeostasis	4,85E-03	1,05E-02	935	20	2,1%
GO:0044085	cellular component biogenesis	3,60E-08	1,64E-07	3285	70	2,1%
GO:0009605	response to external stimulus	2,60E-05	8,32E-05	2471	50	2,0%
GO:0065009	regulation of molecular function	7,39E-06	2,49E-05	3193	62	1,9%
GO:0016043	cellular component organization	4,38E-13	4,00E-12	6387	124	1,9%
GO:0008219	cell death	4,27E-04	1,14E-03	2191	42	1,9%
GO:0048856	anatomical structure development	3,90E-10	2,63E-09	5773	109	1,9%
GO:0007275	multicellular organism development	1,81E-08	8,91E-08	5275	98	1,9%
GO:0048869	cellular developmental process	1,09E-05	3,58E-05	4265	76	1,8%
GO:0006807	nitrogen compound metabolic process	6,04E-15	9,66E-14	10136	170	1,7%
GO:0051716	cellular response to stimulus	7,34E-08	3,13E-07	7448	123	1,7%
GO:0044237	cellular metabolic process	3,74E-14	3,99E-13	10598	173	1,6%
GO:0071704	organic substance metabolic process	2,77E-14	3,22E-13	10986	177	1,6%
GO:0044238	primary metabolic process	4,79E-13	4,09E-12	10624	171	1,6%
GO:0007165	signal transduction	5,14E-05	1,53E-04	6037	96	1,6%
GO:0007154	cell communication	9,51E-05	2,77E-04	6538	101	1,5%
GO:0009058	biosynthetic process	3,50E-04	9,73E-04	6163	94	1,5%
GO:0050789	regulation of biological process	4,45E-07	1,63E-06	11499	165	1,4%

Supplementary table 1: Gene Ontology biological processes highlighted in extracellular proteins in E6-E8 cSG^{cm} and cDRG^{cm}. Hypergeometric statistical test, $p<0.05$. Supplementary table 1 supports data presented in Supplementary Figure 3a.

Supplementary Table 2

term_goid	term_name	p-value	q-value	Effective_size	Nb of items in query	Ratio
**Common to extracellular proteins in E6-E8-cSG^{cm} & cDRG^{cm} and E15-mSG^{cm} & mDRG^{cm}**						
GO:0032963	collagen metabolic process	4,59E-11	8,00E-09	97	12	12,4%
GO:0006457	protein folding	3,52E-04	7,40E-16	226	25	11,1%
GO:1990748	cellular detoxification	6,63E-06	1,83E-05	109	9	8,3%
GO:0019882	antigen processing and presentation	6,12E-07	1,40E-04	176	10	5,7%
GO:0001503	ossification	1,49E-15	2,00E-06	372	18	4,8%
GO:0050817	coagulation	5,61E-18	1,17E-05	342	16	4,7%
GO:0030029	actin filament-based process	1,95E-03	2,28E-10	726	33	4,5%
GO:0007163	establishment or maintenance of cell polarity	1,02E-03	2,13E-03	208	9	4,3%
GO:0044419	interspecies interaction between organisms	6,66E-04	1,22E-10	833	36	4,3%
GO:0032940	secretion by cell	9,98E-09	1,39E-16	1489	61	4,1%
GO:0007155	cell adhesion	3,87E-11	1,68E-14	1387	55	4,0%
GO:0001775	cell activation	1,83E-12	1,68E-14	1432	56	3,9%
GO:0007568	aging	9,44E-04	8,56E-04	307	12	3,9%
GO:0048646	anatomical structure formation involved in mor	4,23E-12	5,61E-11	1075	42	3,9%
GO:0008037	cell recognition	5,42E-05	4,24E-03	231	9	3,9%
GO:0002252	immune effector process	6,27E-03	6,92E-13	1284	50	3,9%
GO:0090130	tissue migration	4,04E-06	1,53E-03	284	11	3,9%
GO:0048589	developmental growth	1,40E-15	2,09E-06	631	24	3,8%
GO:0045321	leukocyte activation	2,43E-22	2,50E-11	1281	47	3,7%
GO:0009056	catabolic process	1,32E-12	1,51E-20	2542	89	3,5%
GO:0009628	response to abiotic stimulus	6,58E-07	1,37E-08	1148	39	3,4%
GO:0055114	oxidation-reduction process	7,81E-14	3,65E-07	991	33	3,3%
GO:0009653	anatomical structure morphogenesis	1,25E-22	4,35E-17	2554	83	3,2%
GO:0016049	cell growth	2,27E-11	1,30E-03	467	15	3,2%
GO:0009719	response to endogenous stimulus	1,40E-18	2,00E-10	1622	52	3,2%
GO:0006928	movement of cell or subcellular component	1,31E-10	1,13E-11	2074	63	3,0%
GO:0006955	immune response	8,86E-14	5,00E-12	2252	67	3,0%
GO:0048870	cell motility	3,42E-09	3,64E-08	1618	47	2,9%
GO:0051674	localization of cell	1,03E-07	3,64E-08	1618	47	2,9%
GO:0044281	small molecule metabolic process	9,98E-09	6,25E-10	2006	58	2,9%
GO:0019725	cellular homeostasis	9,96E-12	3,83E-04	935	25	2,7%
GO:0065008	regulation of biological quality	3,29E-07	8,43E-16	3910	103	2,6%
GO:0042221	response to chemical	1,78E-09	1,83E-18	4644	120	2,6%
GO:0006950	response to stress	6,94E-09	7,32E-13	3958	97	2,5%
GO:0008219	cell death	5,43E-04	3,03E-06	2191	52	2,4%
GO:0016043	cellular component organization	9,37E-15	1,51E-20	6387	149	2,3%
GO:0048869	cellular developmental process	1,45E-06	8,62E-12	4265	99	2,3%
GO:0044085	cellular component biogenesis	9,79E-07	2,69E-08	3285	75	2,3%
GO:0009605	response to external stimulus	7,26E-13	4,39E-06	2471	56	2,3%
GO:0048856	anatomical structure development	6,11E-07	2,94E-15	5773	129	2,2%
GO:0051641	cellular localization	1,55E-04	1,13E-06	2867	64	2,2%
GO:0007275	multicellular organism development	6,62E-06	2,35E-12	5275	115	2,2%
GO:0033036	macromolecule localization	4,76E-17	7,34E-06	3036	64	2,1%
GO:0051234	establishment of localization	3,22E-13	1,23E-09	5224	107	2,0%
GO:0065009	regulation of molecular function	4,43E-20	1,83E-05	3193	65	2,0%
GO:0051716	cellular response to stimulus	1,10E-15	7,60E-09	7448	134	1,8%
GO:0007165	signal transduction	2,48E-06	2,00E-06	6037	108	1,8%
GO:0006807	nitrogen compound metabolic process	1,20E-13	1,52E-14	10136	180	1,8%
GO:0044237	cellular metabolic process	1,09E-05	8,93E-14	10598	183	1,7%
GO:0044238	primary metabolic process	9,10E-15	9,32E-13	10624	181	1,7%
GO:0071704	organic substance metabolic process	1,81E-10	8,93E-14	10986	187	1,7%
GO:0007154	cell communication	2,78E-10	2,93E-05	6538	110	1,7%
GO:0009058	biosynthetic process	1,90E-16	2,91E-04	6163	101	1,6%
GO:0050789	regulation of biological process	1,15E-04	8,29E-10	11499	183	1,6%
**Only in extracellular proteins in E15-mSG^{cm} & mDRG^{cm}**						
GO:0035036	sperm-egg recognition	3,58E-17	1,18E-04	54	6	11,1%
GO:0021700	developmental maturation	5,75E-03	1,30E-02	276	9	3,3%
**Only extracellular proteins in E6-E8-cSG^{cm} & cDRG^{cm}**						
GO:0044706	multi-multicellular organism process	3,88E-03	8,70E-03	222	8	3,6%
GO:0061919	process utilizing autophagic mechanism	9,33E-03	1,99E-02	484	12	2.5%

Supplementary table 2: Gene Ontology biological processes highlighted in extracellular proteins in E15 mSG^{cm} and mDRG^{cm}. Hypergeometric statistical test, $p<0.05$. Supplementary table 2 supports data presented in Figure 2c.

Supplementary Table 3

GENE	Primary references
APP	Rice 2012 (1)
BCL2L1	Cheng 2007 (2)
BMP1	Inomata 2008 (3)
CDH10	Liu 2006 (4), Lee 2007 (5)
CDH6	Liu 2006 (4), Lee 2007 (5)
CDH8	Liu 2006 (4), Lee 2007 (5)
CHRD	Inomata 2008 (3)
DISC1	Rice 2012 (1)
DLG4/PSD95	Sultana 2014 (6)
DTNB	Veroni 2007 (7)
EDN3	Kwon 2009 (8), Mica 2013 (9)
GRIA2	Sultana 2014 (6), Tang 2020 (10)
LGALS3	Block 2011 (11)
LMAN1L	Liu 2006 (4), Lee 2007 (5)
OLFM1	Zeng 2005 (12), Anholt 2014 (13)
OLFM2	Zeng 2005 (12), Anholt 2014 (13)
OLFM3	Zeng 2005 (12), Anholt 2014 (13)
OLFML1	Zeng 2005 (12), Anholt 2014 (13)
OLFML2A	Zeng 2005 (12), Anholt 2014 (13)
OLFML2B	Zeng 2005 (12), Anholt 2014 (13)
OLFML3	Zeng 2005 (12), Anholt 2014 (13)
RTN4R	Nakaya 2012 (14)
SNAP25	Nakaya 2017 (15)
STX11	Tomarev 2009 (16)
SYP	Nakaya 2013 (17)
TENM4	Nakaya 2013 (17); Del Toro 2020 (18)
TLL1	Inomata 2008 (3)
VAMP2	Nakaya 2017 (15)
WASF1	Cheng 2007 (2)

Supplementary table 3: OLFM-related gene set and corresponding primary references from the litterature. Supplementary table 3 supports data presented in Figure 3f.

Supplementary Table 4

	Sample origin	Stage	Biopsy event
#NB1	Adrenal primary tumor	4	Post-chemotherapy
#NB2	Abdominal primary tumor	4	Post-chemotherapy
#NB3	Bone Marrow aspirate (90% tumor cells)	4	Relapse

Supplementary table 4: Listing of NB patient samples used to performed cell aggregation assays and *in ovo* graft experiments.

Supplementary Table 5

NAME	DESCRIPTION	HGNC ID
ADAMTS20	ADAM metallopeptidase with thrombospondin type 1 motif 20	HGNC: 17178
APBB2	amyloid beta precursor protein binding family B member 2	HGNC: 582
ASS1	argininosuccinate synthase 1	HGNC: 758
ATF5	activating transcription factor 5	HGNC: 790
CREB3L1	cAMP responsive element binding protein 3 like 1	HGNC: 18856
ELF4	E74 like ETS transcription factor 4	HGNC: 3319
ERFE	erythroferrone	HGNC: 26727
EXPH5	exophilin 5	HGNC: 30578
FUT1	fucosyltransferase 1 (H blood group)	HGNC: 4012
GALNT18	polypeptide N-acetylgalactosaminyltransferase 18	HGNC: 30488
GYPC	glycophorin C (Gerbich blood group)	HGNC: 4704
HIP1	huntingtin interacting protein 1	HGNC: 4913
HMOX1	heme oxygenase 1	HGNC: 5013
INHBE	inhibin subunit beta E	HGNC: 24029
KCNK1	potassium two pore domain channel subfamily K member 1	HGNC: 6272
KLHDC7B	kelch domain containing 7B	HGNC: 25145
KRT18	keratin 18	HGNC: 6430
MEIOC	meiosis specific with coiled-coil domain	HGNC: 26670
MKI67	marker of proliferation Ki-67	HGNC: 7107
MOGAT1	monoacylglycerol O-acyltransferase 1	HGNC: 18210
NFE2L1	nuclear factor, erythroid 2 like 1	HGNC: 7781
NSUN7	NOP2/Sun RNA methyltransferase family member 7	HGNC: 25857
NXPH4	neurexophilin 4	HGNC: 8078
PDE4A	phosphodiesterase 4A	HGNC: 8780
PHGDH	phosphoglycerate dehydrogenase	HGNC: 8923
PIM1	Pim-1 proto-oncogene, serine/threonine kinase	HGNC: 8986
RAB29	RAB29, member RAS oncogene family	HGNC: 9789
RAD54B	RAD54 homolog B	HGNC: 17228
RAI14	retinoic acid induced 14	HGNC: 14873
RTN4R	reticulon 4 receptor	HGNC: 18601
SHMT2	serine hydroxymethyltransferase 2	HGNC: 10852
SIX4	SIX homeobox 4	HGNC: 10890
SLC25A16	solute carrier family 25 member 16	HGNC: 10986
SLC9A7	solute carrier family 9 member A7	HGNC: 17123
SPHK1	sphingosine kinase 1	HGNC: 11240
TLL1	tolloid like 1	HGNC: 11843
TNFRSF10B	TNF receptor superfamily member 10b	HGNC: 11905
TTC39B	tetratricopeptide repeat domain 39B	HGNC: 23704
UBTD1	ubiquitin domain containing 1	HGNC: 25683
VASH2	vasohibin 2	HGNC: 25723

Supplementary table 5: Genes upregulated in «Primary tumor cell escape» gene set. Genes highlighted in blue are found upregulated in metastatic forms of neural crest-derived cancers.

Supplementary Table 5 (continued)

NAME	DESCRIPTION	HGNC ID
ANK1	ankyrin 1	HGNC: 492
ARHGEF3	Rho guanine nucleotide exchange factor 3	HGNC: 683
BMPR1B	bone morphogenetic protein receptor type 1B	HGNC: 1077
BTBD3	BTB domain containing 3	HGNC: 15854
C1orf216	chromosome 1 open reading frame 216	HGNC: 26800
C3orf62	chromosome 3 open reading frame 62	HGNC: 24771
CACNA2D2	calcium voltage-gated channel auxiliary subunit alpha2delta 2	HGNC: 1400
CAMK2A	calcium/calmodulin dependent protein kinase II alpha	HGNC: 1460
CHRNA3	cholinergic receptor nicotinic alpha 3 subunit	HGNC: 1957
CYB561	cytochrome b561	HGNC: 2571
DBH	dopamine beta-hydroxylase	HGNC: 2689
DUSP26	dual specificity phosphatase 26	HGNC: 28161
EFR3A	EFR3 homolog A	HGNC: 28970
EPC2	enhancer of polycomb homolog 2	HGNC: 24543
FAM167A	family with sequence similarity 167 member A	HGNC: 15549
GFRA3	GDNF family receptor alpha 3	HGNC: 4245
GPR83	G protein-coupled receptor 83	HGNC: 4523
HECW2	HECT, C2 and WW domain containing E3 ubiquitin protein ligase 2	HGNC: 29853
HTR2A	5-hydroxytryptamine receptor 2A	HGNC: 5293
ING4	inhibitor of growth family member 4	HGNC: 19423
LRRTM2	leucine rich repeat transmembrane neuronal 2	HGNC: 19409
MEG3	maternally expressed 3	HGNC: 14575
NBPF1	NBPF member 1	HGNC: 26088
NT5E	5'-nucleotidase ecto	HGNC: 8021
PAPPA	pappalysin 1	HGNC: 8602
PCDHB16	protocadherin beta 16	HGNC: 14546
PCSK6	proprotein convertase subtilisin/kexin type 6	HGNC: 8569
PEAR1	platelet endothelial aggregation receptor 1	HGNC: 33631
PIK3R1	phosphoinositide-3-kinase regulatory subunit 1	HGNC: 8979
PLK2	polo like kinase 2	HGNC: 19699
PRKAR1B	protein kinase cAMP-dependent type I regulatory subunit beta	HGNC: 9390
PTBP2	polypyrimidine tract binding protein 2	HGNC: 17662
PTS	6-pyruvoyltetrahydropterin synthase	HGNC: 9689
RIN2	Ras and Rab interactor 2	HGNC: 18750
SAMD9	sterile alpha motif domain containing 9	HGNC: 1348
SCML4	Scm polycomb group protein like 4	HGNC: 21397
SLC18A1	solute carrier family 18 member A1	HGNC: 10934
SLC44A5	solute carrier family 44 member 5	HGNC: 28524
SNAP25	synaptosome associated protein 25	HGNC: 11132
SORBS2	sorbin and SH3 domain containing 2	HGNC: 24098
SPRY2	sprouty RTK signaling antagonist 2	HGNC: 11270
SPRY4	sprouty RTK signaling antagonist 4	HGNC: 15533
SRSF8	serine and arginine rich splicing factor 8	!"#$%&'()**
STEAP3	STEAP3 metalloreductase	HGNC: 24592
STMN3	stathmin 3	HGNC: 15926
STS	steroid sulfatase	HGNC: 11425
TCTA	T cell leukemia translocation altered	HGNC: 11692
TIMP3	TIMP metallopeptidase inhibitor 3	HGNC: 11822
TRIL	TLR4 interactor with leucine rich repeats	HGNC: 22200
TRPM3	transient receptor potential cation channel subfamily M member 3	HGNC: 17992
UTRN	utrophin	HGNC: 12635
ZMIZ1	zinc finger MIZ-type containing 1	HGNC: 16493
ZNF608	zinc finger protein 608	HGNC: 29238

Supplementary table 5 (continued): Genes downregulated in «Primary tumor cell escape» gene set. Genes highlighted in blue are found downregulated in metastatic forms of neural crest-derived cancers.

Supplementary References

1. Rice, H. C. *et al.* Pancortins interact with amyloid precursor protein and modulate cortical cell migration. *Development* **139**, 3986–96 (2012).
2. Cheng, A. *et al.* Pancortin-2 interacts with WAVE1 and Bcl-xL in a mitochondria-associated protein complex that mediates ischemic neuronal death. *J. Neurosci.* **27**, 1519–28 (2007).
3. Inomata, H., Haraguchi, T. & Sasai, Y. Robust stability of the embryonic axial pattern requires a secreted scaffold for chordin degradation. *Cell* **134**, 854–65 (2008).
4. Liu, W., Chen, L., Zhu, J. & Rodgers, G. P. The glycoprotein hGC-1 binds to cadherin and lectins. *Exp. Cell Res.* **312**, 1785–97 (2006).
5. Lee, H.-S. & Tomarev, S. I. Optimedin induces expression of N-cadherin and stimulates aggregation of NGF-stimulated PC12 cells. *Exp. Cell Res.* **313**, 98–108 (2007).
6. Sultana, A. *et al.* Deletion of olfactomedin 2 induces changes in the AMPA receptor complex and impairs visual, olfactory, and motor functions in mice. *Exp. Neurol.* **261**, 802–11 (2014).
7. Veroni, C. *et al.* β-dystrobrevin, a kinesin-binding receptor, interacts with the extracellular matrix components pancortins. *J. Neurosci. Res.* **85**, 2631–2639 (2007).
8. Kwon, H.-S., Lee, H.-S., Ji, Y., Rubin, J. S. & Tomarev, S. I. Myocilin is a modulator of Wnt signaling. *Mol. Cell. Biol.* **29**, 2139–54 (2009).
9. Mica, Y., Lee, G., Chambers, S. M., Tomishima, M. J. & Studer, L. Modeling neural crest induction, melanocyte specification, and disease-related pigmentation defects in hESCs and patient-specific iPSCs. *Cell Rep.* **3**, 1140–52 (2013).
10. Tang, S. *et al.* Olfactomedin-3 Enhances Seizure Activity by Interacting With AMPA Receptors in Epilepsy Models. *Front. cell Dev. Biol.* **8**, 722 (2020).
11. Block, A. S., Saraswati, S., Lichti, C. F., Mahadevan, M. & Diekman, A. B. Co-purification of Mac-2 binding protein with galectin-3 and association with prostasomes in human semen. *Prostate* **71**, 711–21 (2011).
12. Zeng, L.-C., Han, Z.-G. & Ma, W.-J. Elucidation of subfamily segregation and intramolecular coevolution of the olfactomedin-like proteins by comprehensive phylogenetic analysis and gene expression pattern assessment. *FEBS Lett.* **579**, 5443–53 (2005).
13. Anholt, R. R. H. Olfactomedin proteins: central players in development and disease. *Front. cell Dev. Biol.* **2**, 6 (2014).
14. Nakaya, N., Sultana, A., Lee, H.-S. & Tomarev, S. I. Olfactomedin 1 Interacts with the Nogo A Receptor Complex to Regulate Axon Growth. *J. Biol. Chem.* **287**, 37171–37184 (2012).
15. Nakaya, N., Sultana, A. & Tomarev, S. I. Impaired AMPA receptor trafficking by a double knockout of zebrafish olfactomedin1a/b. *J. Neurochem.* **143**, 635–644 (2017).
16. Tomarev, S. I. & Nakaya, N. Olfactomedin domain-containing proteins: possible mechanisms of action and functions in normal development and pathology. *Mol. Neurobiol.* **40**, 122–38 (2009).
17. Nakaya, N. *et al.* Deletion in the N-terminal half of olfactomedin 1 modifies its interaction with synaptic proteins and causes brain dystrophy and abnormal behavior in mice. *Exp. Neurol.* **250**, 205–18 (2013).
18. Del Toro, D. *et al.* Structural Basis of Teneurin-Latrophilin Interaction in Repulsive Guidance of Migrating Neurons. *Cell* **180**, 323-339.e19 (2020).
19. Vallin, J., Girault, J. M., Thiery, J. P. & Broders, F. Xenopus cadherin-11 is expressed in different populations of migrating neural crest cells. *Mech. Dev.* **75**, 171–4 (1998).
20. McCusker, C., Cousin, H., Neuner, R. & Alfandari, D. Extracellular cleavage of cadherin-11 by ADAM metalloproteases is essential for Xenopus cranial neural crest cell migration. *Mol. Biol. Cell* **20**, 78–89 (2009).
21. Hatta, K., Nature, M. T.- & 1986, undefined. Expression of N-cadherin adhesion molecules associated with early morphogenetic events in chick development. *nature.com*
22. Akitaya, T. & Bronner-Fraser, M. Expression of cell adhesion molecules during initiation and cessation of neural crest cell migration. *Dev. Dyn.* **194**, 12–20 (1992).
23. Nakagawa, S. & Takeichi, M. Neural crest emigration from the neural tube depends on regulated cadherin expression. *Development* **125**, 2963–2971 (1998).
24. Lallier, T., Leblanc, G., Artinger, K. B. & Bronner-Fraser, M. Cranial and trunk neural crest cells use different mechanisms for attachment to extracellular matrices. *Development* **116**, 531–41 (1992).
25. Wang, X. & Astrof, S. Neural crest cell-autonomous roles of fibronectin in cardiovascular development. *Development* **143**, 88–100 (2016).
26. Ruiz-Perera, L. M., Arruti, C. & Zolessi, F. R. Early phosphorylation of MARCKS at Ser25 in

migrating precursor cells and differentiating peripheral neurons. *Neurosci. Lett.* **544**, 5–9 (2013).

27. Cai, D. H., Vollberg, T. M., Hahn-Dantona, E., Quigley, J. P. & Brauer, P. R. MMP-2 expression during early avian cardiac and neural crest morphogenesis. *Anat. Rec.* **259**, 168–79 (2000).
28. Duong, T. D. & Erickson, C. A. MMP-2 plays an essential role in producing epithelial-mesenchymal transformations in the avian embryo. *Dev. Dyn.* **229**, 42–53 (2004).
29. Moreno, T. A. & Bronner-Fraser, M. The secreted glycoprotein Noelin-1 promotes neurogenesis in Xenopus. *Dev. Biol.* **240**, 340–60 (2001).
30. Barembaum, M., Moreno, T. A., LaBonne, C., Sechrist, J. & Bronner-Fraser, M. Noelin-1 is a secreted glycoprotein involved in generation of the neural crest. *Nat. Cell Biol.* **2**, 219–225 (2000).
31. Debby-Brafman, A., Burstyn-Cohen, T., Klar, A. & Kalcheim, C. F-Spondin, expressed in somite regions avoided by neural crest cells, mediates inhibition of distinct somite domains to neural crest migration. *Neuron* **22**, 475–88 (1999).
32. Kawakami, T. *et al.* Transforming growth factor beta1 regulates melanocyte proliferation and differentiation in mouse neural crest cells via stem cell factor/KIT signaling. *J. Invest. Dermatol.* **118**, 471–8 (2002).
33. Li, X. *et al.* Differentiation of Neural Crest Stem Cells in Response to Matrix Stiffness and TGF-β1 in Vascular Regeneration. *Stem Cells Dev.* **29**, 249–256 (2020).
34. Cantemir, V., Cai, D. H., Reedy, M. V & Brauer, P. R. Tissue inhibitor of metalloproteinase-2 (TIMP-2) expression during cardiac neural crest cell migration and its role in proMMP-2 activation. *Dev. Dyn.* **231**, 709–19 (2004).
35. Perris, R. *et al.* Inhibitory effects of PG-H/aggrecan and PG-M/versican on avian neural crest cell migration. *FASEB J.* **10**, 293–301 (1996).
36. Dutt, S., Kléber, M., Matasci, M., Sommer, L. & Zimmermann, D. R. Versican V0 and V1 guide migratory neural crest cells. *J. Biol. Chem.* **281**, 12123–31 (2006).
37. Dong, R. *et al.* Single-Cell Characterization of Malignant Phenotypes and Developmental Trajectories of Adrenal Neuroblastoma. *Cancer Cell* **38**, 716-733.e6 (2020).
38. Jansky, S. *et al.* Single-cell transcriptomic analyses provide insights into the developmental origins of neuroblastoma. *Nat. Genet.* (2021). doi:10.1038/s41588-021-00806-1
