# Supplementary material for: Environmental cues from neural crest derivatives act as metastatic triggers in an embryonic neuroblastoma model
Source: Nat Commun. 2022 May 10;13:2549. doi: 10.1038/s41467-022-30237-3 (PMC9091272; doi:10.1038/s41467-022-30237-3)
Supplement: Supplementary file 3 — Description of Additional Supplementary Files [file 41467_2022_30237_MOESM3_ESM.pdf]

Description of Additional Supplementary Files

Supplementary Data 1: Restricted (NOR;MES) and extended (NOR-all; MES-all) gene sets curated from Boeva et al (2017); Van Groningen et al (2017); Dong et al (2020) and Jansky et al (2021)
