# Supplementary material for: Environmental cues from neural crest derivatives act as metastatic triggers in an embryonic neuroblastoma model
Source: Nat Commun. 2022 May 10;13:2549. doi: 10.1038/s41467-022-30237-3 (PMC9091272; doi:10.1038/s41467-022-30237-3)
Supplement: Supplementary file 5 — Reporting Summary [file 41467_2022_30237_MOESM5_ESM.pdf]

nature portfolio

Corresponding author(s): Valérie Castellani and Céline Delloye-Bourgeois

Last updated by author(s): 17/03/22

Reporting Summary

Nature Portfolio wishes to improve the reproducibility of the work that we publish. This form provides structure for consistency and transparency in reporting. For further information on Nature Portfolio policies, see our Editorial Policies and the Editorial Policy Checklist.

Statistics

For all statistical analyses, confirm that the following items are present in the figure legend, table legend, main text, or Methods section.

n/a	Confirmed	
☐	☒	The exact sample size (n) for each experimental group/condition, given as a discrete number and unit of measurement
☐	☒	A statement on whether measurements were taken from distinct samples or whether the same sample was measured repeatedly
☐	☒	The statistical test(s) used AND whether they are one- or two-sided<br>*Only common tests should be described solely by name; describe more complex techniques in the Methods section.*
☒	☐	A description of all covariates tested
☐	☒	A description of any assumptions or corrections, such as tests of normality and adjustment for multiple comparisons
☐	☒	A full description of the statistical parameters including central tendency (e.g. means) or other basic estimates (e.g. regression coefficient) AND variation (e.g. standard deviation) or associated estimates of uncertainty (e.g. confidence intervals)
☐	☒	For null hypothesis testing, the test statistic (e.g. F, t, r) with confidence intervals, effect sizes, degrees of freedom and P value noted<br>*Give P values as exact values whenever suitable.*
☒	☐	For Bayesian analysis, information on the choice of priors and Markov chain Monte Carlo settings
☒	☐	For hierarchical and complex designs, identification of the appropriate level for tests and full reporting of outcomes
☒	☐	Estimates of effect sizes (e.g. Cohen's d, Pearson's r), indicating how they were calculated

Our web collection on statistics for biologists contains articles on many of the points above.

Software and code

Policy information about availability of computer code

Data collection	Imaging devices:<br>- Fluorescence stereomicroscope Leica MZ10F<br>- QImaging camera MicroPublisher 5.0 (GTVision)<br>- Confocal microscope Olympus, FV1000, X81<br>- Light sheet Ultramicroscope, Miltenyi Biotec<br>- ImSpector Pro (7.1.15) LaVision BioTec GmbH<br><br>RNASeq device:<br>- Bioanalyzer 2100, Agilent<br>- NextSeq500 illumina<br><br>Mass spectrometry devices:<br>- UltiMate 3000 and LTQ-Orbitrap Velos Pro, Thermo Scientific<br>- Xcalibur, Thermo Scientific
Data analysis	Statistics:<br>- Prism 9.0e (Graphpad)<br><br>Image analysis:<br>- Image J (1.53a)<br>- Arivis Vision4D (3.1.3)

Proteomic tools:
- Mascot (v2.6)
-Proline (v2.1.2)

Bioinformatic tools:
- Bcl2fastq v2.17.1.14
-CutAdapt v1.9.1
-HTSeq-count software (0.11.3)
-TopHat v.2.1.0
-DESeq2 (3.11)
-GSEA (www.gsea-msigdb.org)
-ToppGene (https://toppgene.cchmc.org)
-CPDB (http://cpdb.molgen.mpg.de/)
-R2 Genomics Analysis and Visualization platform (http://r2.amc.nl)
-Seurat package (R)
-DoubletFinder (v2.0.3) (R)
-AddModuleScore (in Seurat)

For manuscripts utilizing custom algorithms or software that are central to the research but not yet described in published literature, software must be made available to editors and reviewers. We strongly encourage code deposition in a community repository (e.g. GitHub). See the Nature Portfolio guidelines for submitting code & software for further information.

Data

Policy information about availability of data

All manuscripts must include a data availability statement. This statement should provide the following information, where applicable:

- Accession codes, unique identifiers, or web links for publicly available datasets
- A description of any restrictions on data availability
- For clinical datasets or third party data, please ensure that the statement adheres to our policy

Raw and analyzed bulk RNASeq data generated in the present article have been deposited in the Gene Expression Omnibus (GEO) under accession number GSE169280 (https://www.ncbi.nlm.nih.gov/geo/query/acc.cgi?acc=GSE169280).
The mass spectrometry proteomics data have been deposited to the ProteomeXchange Consortium via the PRIDE partner repository with the dataset identifier PXD027499.

Bulk RNA-seq data of patient cohorts were obtained from:
- neuroblastoma GSE85047: https://www.ncbi.nlm.nih.gov/geo/query/acc.cgi?acc=GSE85047
- neuroblastoma GSE62564: https://www.ncbi.nlm.nih.gov/geo/query/acc.cgi?acc=GSE62564
- neuroblastoma GSE45480: https://www.ncbi.nlm.nih.gov/geo/query/acc.cgi?acc=GSE45480
- neuroblastoma GSE120572: https://www.ncbi.nlm.nih.gov/geo/query/acc.cgi?acc=GSE120572
- neuroblastoma E-MTAB-8248: https://www.ebi.ac.uk/arrayexpress/experiments/E-MTAB-8248/
- melanoma GSE65904: https://www.ncbi.nlm.nih.gov/geo/query/acc.cgi?acc=GSE65904
- pheochromocytoma GSE67066: https://www.ncbi.nlm.nih.gov/geo/query/acc.cgi?acc=GSE67066
- colorectal cancer GSE39582: https://www.ncbi.nlm.nih.gov/geo/query/acc.cgi?acc=GSE39582
- pancreatic cancer TCGA-PAAD: https://portal.gdc.cancer.gov/projects/TCGA-PAAD
- breast cancer GSE102484: https://www.ncbi.nlm.nih.gov/geo/query/acc.cgi?acc=GSE102484

Single cell RNASeq data of fetal adrenal medullary gland were obtained from Jansky et al., 2021: EGA ID: EGAS00001004388 [https://ega-archive.org/studies/EGAS00001004388]
Single cell RNASeq data of NB tumors were obtained from Dong et al., 2020: GEO ID: GSE137804 [https://www.ncbi.nlm.nih.gov/geo/query/acc.cgi?acc=GSE137804]

Other data that support the findings of the study are available from the corresponding authors upon reasonnable request.

Field-specific reporting

Please select the one below that is the best fit for your research. If you are not sure, read the appropriate sections before making your selection.

- [x] Life sciences
- [ ] Behavioural & social sciences
- [ ] Ecological, evolutionary & environmental sciences

For a reference copy of the document with all sections, see nature.com/documents/nr-reporting-summary-flat.pdf

Life sciences study design

All studies must disclose on these points even when the disclosure is negative.

Sample size	No statistical tests or analyses were used to predetermine sample size; however, our sample sizes are similar to those reported in previous publications (Delloye-Bourgeois et al., Cancer Cell, 2017). Statistical treatment of the data could be performed for each dataset presented in the study and choice of statistical tests was rationalized with a dedicated software (Prism 9.0e).

Data exclusions	For cell aggregation assays, hanging drops that had partially flowed were excluded due to systematic impaired cell aggregation.<br>For in vivo experiments on avian embryos, dead embryos by the time of harvest were not included in tumor/metastases analyses.
Replication	The exact number of replication is mentionned for each dataset in the figures legends.<br>In vitro experiments (aggregation assays, migration and invasion assays) were repeated in at least 3 independent experiments with as least 3 biological replicates for each experimental condition.<br>In vivo experiments (analyses of NB tumors and metastases in avian embryos engrafted with cell lines or patient samples) were analyzed in groups of embryos containing at least 7 subjects per condition.<br>Bulk RNASeq analyses were performed in duplicates for each experimental condition.<br>Proteomic analyses were performed in simplicate but each analyzed conditioned media was obtained from 3 independent dissections.
Randomization	Control and experimental conditions were treated without any distinction in all experiments. For in vivo experiments, embryos were allocated to experimental groups randomly.
Blinding	For in vitro aggregation assays and migration/invasion assays, data collection and analysis was done in blind. Experiments on chick embryos were analyzed in blind regarding each experimental group but investigators were not blinded for data collection as it would have been impossible to adequately assign each embryo to the correct experimental group. RNASeq data generated here or from previous studies and proteomic data were not collected nor analyzed in blind as the objective was to define objectively the differences between experimental groups.

Reporting for specific materials, systems and methods

We require information from authors about some types of materials, experimental systems and methods used in many studies. Here, indicate whether each material, system or method listed is relevant to your study. If you are not sure if a list item applies to your research, read the appropriate section before selecting a response.

Materials & experimental systems

n/a	Involved in the study
☐	☒ Antibodies
☐	☒ Eukaryotic cell lines
☒	☐ Palaeontology and archaeology
☐	☒ Animals and other organisms
☐	☒ Human research participants
☒	☐ Clinical data
☒	☐ Dual use research of concern

Methods

n/a	Involved in the study
☒	☐ ChIP-seq
☒	☐ Flow cytometry
☒	☐ MRI-based neuroimaging

Antibodies

Antibodies used	- Rabbit anti-Green Fluorescent Protein (GFP) Polyclonal Antibody Thermo Fisher Scientific Cat# A-11122; RRID: AB_221569<br>- Mouse anti-NEFM monoclonal Antibody (RMO-270) Thermo Fisher Scientific Cat# 13-0700; RRID: AB_2532998<br>- Rabbit anti-phospho H3 IgG antibody Cell signaling Cat# 3377S<br>- Sheep anti-Olfactomedin-1/Noelin-1 Antibody R&D Systems Cat #AF4636<br>- Mouse anti-Mitochondria alpha antibody Millipore/ Sigma MAB1273 ; RRID: AB_94052<br>- Mouse anti-HNK1 IgM antibody Hybridoma Bank Cat # 3H5 ; RRID : AB_2314644<br>- Rabbit anti-OLFM1 Antibody; Atlas Antibodies Cat# HPA057444, RRID:AB_2683443<br>- Goat anti-hNogoR/RTN4R Antibody, R&D Systems, #AF1208; RRID:AB_2254206<br>- Rabbit anti-GATA3 Antibody; Cell Signaling Technology Cat# 5852, RRID:AB_10835690<br>- Rabbit anti-PHOX2B Antibody, ThermoScientific, #PA5115754<br>- Rabbit anti-c-JUN Antibody; Cell Signaling Technology Cat# 9165, RRID:AB_2130165<br>- Mouse anti-MAML2 Antibody; Sigma-Aldrich Cat# WH0084441M3, RRID:AB_1842365<br>- Mouse anti-RUNX1 Antibody; Santa Cruz Biotechnology Cat# sc-365644, RRID:AB_10843207<br>- Rabbit anti-GAPDH Antibody; Sigma-Aldrich Cat# G9545, RRID:AB_796208<br>- Rabbit anti-chick Phox2b; produced in JF Brunet's lab.
Validation	- Rabbit anti-Green Fluorescent Protein (GFP): the antibody was validated in more than 1303 publications and was validated by the manufacturer specifically for Immunofluorescence: https://www.thermofisher.com/antibody/product/GFP-Antibody-Polyclonal/A-11122<br>- Mouse anti-NEFM monoclonal Antibody: the antibody was validated in more than 54 publications. The manufacturer provides data showing that this Antibody was verified by Knockout to ensure that the antibody binds to the antigen stated, in IHC and immunofluorescence: https://www.thermofisher.com/antibody/product/NEFM-Antibody-clone-RMO-270-Monoclonal/13-0700<br>- Rabbit anti-phospho H3 IgG antibody: the antibody was validated in more than 194 publications and was validated by the manufacturer specifically for Immunofluorescence: https://www.cellsignal.com/products/primary-antibodies/phospho-histone-h3-ser10-d2c8-xp-rabbit-mab/3377<br>- Sheep anti-Olfactomedin-1/Noelin-1 Antibody: the antibody was specifically validated for its function-blocking properties in chick

embryonic tissues in Lencinas et al., Dis Model Mech, 2013;6(0):632-42. Further validations are provided by the manufacturer: https://www.rndsystems.com/products/mouse-olfactomedin-1-noelin-1-antibody_af4636
- Mouse anti-Mitochondria alpha antibody: the antibody was validated in more than 196 publications and was validated by the manufacturer specifically for Immunofluorescence. Human specificity, in particular in Immunofluorescence / Immunohistochemistry applications were performed by the manufacturer and are documented here: https://www.sigmaaldrich.com/FR/fr/product/mm/mab1273?gclid=Cj0KCQjw29CRBhCUARIsAOboZbJcj0uNlWbkhCO7V4KunrOr6Y9Xd0lnScujaSWpiArcv4eezOmfs7saApgXEALw_wcB
- Mouse anti-HNK1 IgM antibody: the antibody was validated in more than 10 publications and was validated by the manufacturer specifically for Immunofluorescence on chick embryonic tissues: https://dshb.biology.uiowa.edu/3H5
- Rabbit anti-OLFM1 Antibody: the antibody was specifically validated by the manufacturer for Immunofluorescence application: https://www.atlasantibodies.com/products/antibodies/primary-antibodies/triple-a-polyclonals/olfm1-antibody-hpa057444/
- Goat anti-NogoR/RTN4R Antibody: the antibody was validated in more than 7 publications and was specifically validated by the manufacturer for its function-blocking properties (blockade of ligand(s)-receptor interaction). The function blocking properties of the antibody were used in Yan et al., J NeuroChem, 2012; Petrinovic et al., Development, 2010. Further validations are provided by the manufacturer: https://www.rndsystems.com/products/human-nogo-receptor-ngr-antibody_af1208
- Rabbit anti-GATA3 Antibody: the antibody was validated in more than 65 publications and was validated by the manufacturer for a range of applications, including Western Blot and Immunofluorescence, notably for human material. Further validations are provided by the manufacturer: https://www.cellsignal.com/products/primary-antibodies/gata-3-d13c9-xp-rabbit-mab/5852
- Rabbit anti-PHOX2B Antibody: the antibody was validated by the manufacturer for Western Blot and Immunofluorescence of human and mouse cells: https://www.fishersci.com/shop/products/phox2b-rabbit-anti-human-mouse-polyclonal-invitrogen/PIPA5115754
- Rabbit anti-c-JUN Antibody: the antibody was validated in more than 718 publications. The manufacturer provides validation data for Western Blot, IHC and Immunofluorescence on human / mouse cells: https://www.cellsignal.com/products/primary-antibodies/c-jun-60a8-rabbit-mab/9165
- Mouse anti-MAML2 Antibody (clone 4A1): the antibody was in 4 publications at least and the manufacturer provides validation data for Western Blot application on human cells: https://www.sigmaaldrich.com/FR/fr/product/sigma/wh0084441m3
- Mouse anti-RUNX1 antibody: the antibody was validated in more than 29 publications and the manufacturer provides validation data for immunofluorescence, IHC and Western Blot in human cells: https://www.scbt.com/fr/p/runx1-antibody-a-2
- Rabbit anti-GAPDH Antibody: the antibody was validated in more than 85 publications and the manufacturer provides validation data for Western Blot and Immunofluorescence: https://www.sigmaaldrich.com/FR/fr/product/sigma/g9545?gclid=Cj0KCQjw29CRBhCUARIsAOboZbJF753IYUdz-yFU1lZ4O7SL1Rgu2ZwcwOC2tXWOJB-Xe92KJrHFu6EaAj-4EALw_wcB
- Rabbit anti-chick Phox2B: the antibody was raised against the C-terminal peptide [(Y)PGGTKGSLVKSGMF] of Phox2b from Pseudopodoces humilis (Tibetan ground-tit), originally the only avian Phox2b gene whose complete open reading frame was correctly predicted from the genome, as judged from the alignment with vertebrate orthologues — and which later turned out identical to the C-terminal peptide encoded by the corrected Phox2b open reading frame for Gallus gallus (Ensembl release 86, Gallus_gallus-5). The antibody was validated for immunofluorescence applications on chick embryo sections of various developmental stages. In the present study, we show a specific labeling of nascent sympathetic ganglia at E5.5.

Eukaryotic cell lines

Policy information about cell lines

Cell line source(s)	IGR-N91 cell line (RRID:CVCL_8883) was given by the team of J. Bénard (Gustave Roussy Institute) and described in Ferrandis and Bénard, 1993.<br>SH-SY5Y cell line was obtained from ATCC (ATCC® CRL-2266™).<br>SHEP cell line was provided by the team of V. Combaret (Centre Léon Bérard): (RRID:CVCL_0524)<br>IGR-N91::GFP and SH-SY5Y::GFP cell lines were previously described in Delloye-Bourgeois et al., Cancer Cell; 2017 and were generated upon lentiviral infection of source cell lines.
Authentication	Cell lines were regularly checked for the expression of key markers by qRT-PCR (MYCN, SEMA3C). Their typical morphology in in vitro culture conditions was also verified at each step of the project.
Mycoplasma contamination	All cell lines were tested negative for mycoplasma contamination.
Commonly misidentified lines (See ICLAC register)	No commonly misidentified cell lines were used in this study.

Animals and other organisms

Policy information about studies involving animals; ARRIVE guidelines recommended for reporting animal research

Laboratory animals	Naked Neck strain embryonated eggs were obtained from a local supplier (Elevage avicole du Grand Buisson, Saint Maurice sur Dargoire, France). Laying hen's sanitary status was regularly checked by the supplier according to French laws.
Wild animals	N/A
Field-collected samples	The study did not involved samples collected from the field.
Ethics oversight	Chick embryos were used within the 10 first days of gestation, stages that do not require approved protocol by ethics committee.

Note that full information on the approval of the study protocol must also be provided in the manuscript.

Human research participants

Policy information about studies involving human research participants

Population characteristics	Stage 4 Neuroblastoma patient samples were obtained from patients treated in Centre Léon Bérard and Hopital Femme-Mère-Enfant (Lyon, France). The only criteria was the stage 4/M status of the tumor sample.<br>- NB#1: Sex M; Age: 147 months<br>- NB#2: Sex F; Age: 48 months<br>- NB#3: Sex F; Age: 40 months
Recruitment	Patients were recruited prospectively, the only criteria was the stage 4/M status of the tumor sample at diagnosis.
Ethics oversight	Studies were authorized by the ethics committees ''Comité de Protection des Personnes Sud-Est IV'' (L07-95 and L12-171), as well as ''Comité de Protection des Personnes de Paris IIe de France I'' (ref 08-11728). Parents provided written consent for tumour banking and future research use according to national regulations.

Note that full information on the approval of the study protocol must also be provided in the manuscript.
